# Glycyrrhizin in patients who failed previous interferon alpha-based therapies: biochemical and histological effects after 52 weeks

**DOI:** 10.1111/j.1365-2893.2011.01579.x

**Published:** 2012-08

**Authors:** M P Manns, H Wedemeyer, A Singer, N Khomutjanskaja, H P Dienes, T Roskams, R Goldin, U Hehnke, H Inoue, the European SNMC Study Group

**Affiliations:** 1 Department of Gastroenterology, Hepatology and Endocrinology, Hannover Medical SchoolHannover, Germany; 2 Minophagen Pharmaceutical Co., Ltd.Tokyo, Japan; 3 PharmaPart GmbHWiesbaden, Germany; 4 Lugansk State Medical University, Department of Infective DiseasesLugansk, Ukraine; 5 Institute of Pathology, University Hospital of CologneCologne, Germany; 6 Department of Pathology, Department of Morphology and Molecular Pathology, University of LeuvenLeuven, Belgium; 7 Imperial College School of MedicineLondon, UK

**Keywords:** ALT, chronic hepatitis C, fibrosis, glycyrrhizin, hepatocellular carcinoma, IFN non-responders, necro-inflammation

## Abstract

Chronic hepatitis C patients often fail to respond to interferon-based therapies. This phase III study aimed at confirming the efficacy and safety of glycyrrhizin in interferon + ribavirin-based therapy non-responders. A randomised, double-blind, placebo-controlled, comparison of glycyrrhizin, administered intravenously 5×/or 3×/week, and 5×/week placebo for 12 weeks to 379 patients, was followed by a randomised, open comparison of glycyrrhizin i.v. 5×/versus 3×/week for 40 weeks. Primary endpoints were: (1) the proportion of patients with ≥50% ALT (alanine aminotransferase) reduction after 12 weeks double-blind phase, and (2) the proportion of patients with improvement of necro-inflammation after 52 weeks as compared with baseline. The proportion of patients with ALT reduction ≥50% after 12 weeks was significantly higher with 5×/week glycyrrhizin (28.7%, *P* < 0.0001) and 3×/week glycyrrhizin (29.0%, *P* < 0.0001) compared with placebo (7.0%). The proportion of patients with improvement in necro-inflammation after 52 weeks was 44.9% with 5×/week and 46.0% with 3×/week, respectively. Glycyrrhizin exhibited a significantly higher ALT reduction compared to placebo after 12 weeks of therapy and an improvement of necro-inflammation and fibrosis after 52-weeks treatment. Generally, glycyrrhizin treatment was well tolerated.

## Introduction

Treatment of hepatitis C virus (HCV) infection has significantly improved over the past 2 decades. However, the current standard of care (SOC), pegylated interferon alpha (peg-IFNα) plus ribavirin (RBV), has its limitations. Sustained virological response (SVR) rates are less than 50% for genotype 1 patients and there is no SOC for non-responders to date [1,2]. In addition, contraindications as well as intolerance to IFN based therapies leave a large number of patients ineligible for this medication. These patients are at risk to develop cirrhosis and its complications, including hepatocellular carcinoma (HCC) [3].

Studies with long-term application of low dose peg-IFNα failed to achieve reduced progression of liver disease. These studies showed that inflammation and fibrosis progression could not be suppressed [4,5]. However, the COPILOT study demonstrated positive effects on portal hypertension [5]. To what extent new direct acting antivirals (DAAs) will meet the expectations for IFN failure or IFN intolerant patients remains uncertain. Response rates to triple therapy with HCV protease inhibitors, peg-IFNα and ribavirin in previous nonresponder patients with liver cirrhosis are still below 20% [6]. Therefore it is legitimate to search for alternative treatment strategies to suppress inflammatory activity and fibrosis progression in non-responders to IFN-based therapies.

Intravenous glycyrrhizin (GL) has been used for more than 30 years in the treatment of liver diseases in Asian countries, mainly in Japan. Studies performed in Asia and Europe showed that administration of GL in patients with chronic hepatitis C (CHC) leads to an improvement of necro-inflammation and liver function tests in a significant proportion of patients. Such effects were also observed in IFN non-responders. It has been demonstrated that there was a dose-dependent effect of GL on elevated ALT levels. The observed decrease of ALT level was rapid, linear and could be maintained in CHC patients receiving at least three injections weekly for varying duration of treatment [7–10]. ALT is a marker of biochemical necro-inflammatory activity of the liver. Persistently high ALT levels are correlated with disease progression leading to complications such as cirrhosis and HCC [11]. The effect of GL is mediated primarily through a suppression of inflammation and through a decreased liver cell injury. The anti-inflammatory activity of GL may be mediated by the direct binding of the molecule to cell membrane components, especially to lipocortin I or to enzymes like phospholipase-A2, which is the initial enzyme in the arachidonic acid metabolic system. GL also directly binds to lipoxygenase, an enzyme to produce the inflammatory chemical mediators. GL selectively inhibits the activation by phosphorylation of these enzymes [12,13]. Furthermore GL and its derivatives are able to inhibit the production of inflammatory chemokines IL-8 and eotaxin 1, which are both potent chemo-attractants to leukocytes during inflammation, and may counteract the expression of those pro-inflammatory chemokines [14]. Long term treatment with GL reduced the incidence of HCC in some studies [7,15–17].

Most studies with GL therapy were performed in Asia so far. In this study the efficacy and safety of GL was evaluated in a 52-weeks treatment of European chronic hepatitis C patients not responding or having contraindications to standard therapy (IFN + RBV or peg-IFNα + RBV).

## Patients and methods

### Patients and study design

**Fig. 1:**
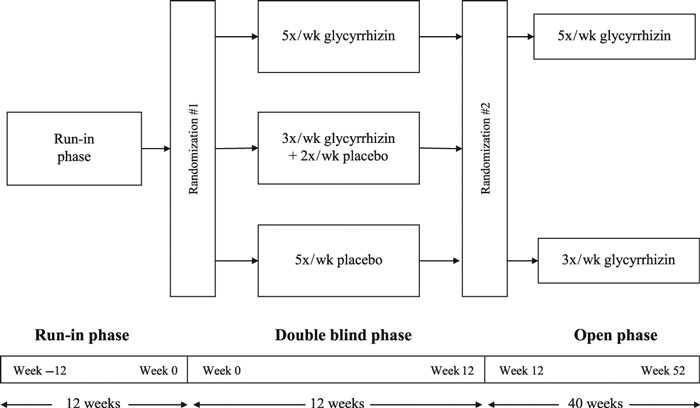
Study flow chart. In this randomised, double-blind, placebo-controlled study, glycyrrhizin was administered intravenously 5×/or 3×/week, or placebo was injected 5×/week for 12 weeks to 379 patients. This double-blind phase was followed by a randomised, open comparison of glycyrrhizin i.v. 5×/versus 3×/week for 40 weeks.

This Phase III study was conducted at 73 centres in 11 European countries from October 2002 to April 2006. Study duration was 52 weeks per patient and consisted of two phases: a 12 weeks double-blind phase followed by an open phase of 40 weeks. Two different regimens of GL were compared to placebo during the double-blind phase (Fig. 1). After completing this phase, all patients were randomised again into the subsequent open phase. The study used a four-stage group sequential adaptive design with sample size adjustments after the planned interim analysis [18,19].

**Fig. 2:**
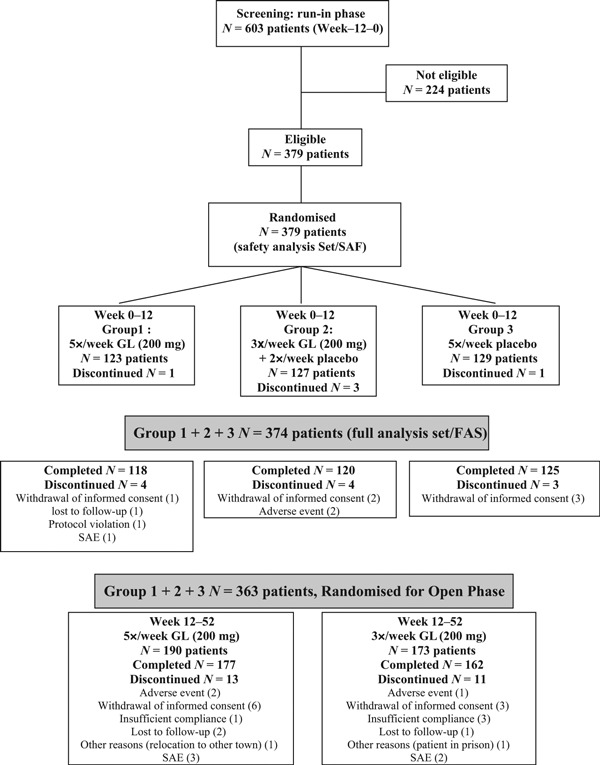
Study design and patient disposition.

Out of 603 screened patients 379 were randomised for the first phase. While 16 patients (4.2%) dropped out from the study during the double-blind phase 363 patients (95.8%) were randomised into the subsequent open phase from Week 13 to Week 52. Another 24 patients (6.6%) terminated the study prematurely, thus 339 patients (93.4%) completed the open phase (Fig. 2).

Demographic data are shown in Table 1. Male and female patients fulfilling the following main inclusion criteria were enrolled: age 18–65 years, positive serum HCV-RNA and documented non response or un-sustained response to treatment with IFN + RBV or peg-IFNα + RBV therapy for at least 3 months. Non-response was defined as positive serum HCV-RNA and abnormal ALT values, with ALT value >1.5× upper limit of normal (ULN). Non response or intolerance was determined by the individual investigator according to standard criteria. Further inclusion criteria were contraindications to IFN therapy, ALT > 2× ULN (22 U/L for male, 17 U/L for female) on at least two occasions within 12 weeks before randomisation, necro-inflammation and fibrosis score positive (score ≥1). Exclusion criteria were: chronic hepatitis B, auto-immune hepatitis, haemochromatosis, Wilson disease, α-1-antitrypsin deficiency, decompensated liver cirrhosis, HCC, fatty liver, cholestasis, serious concomitant disease, antiviral treatment, contraindications to GL or liquorice, alcohol or drug abuse, and female patients with absence of adequate contraceptive measures, pregnancy or breast-feeding.

**Table 1:** Patient characteristics (FAS)

Characteristics	All patients	5×/week GL	3×/week GL + 2×/week Placebo	5×/week Placebo
Total number of patients	*n* = 374	*n* = 122	*n* = 124	*n* = 128
Male (%)	67.9	64.8	63.7	75.0
Age (year) (mean ± sd)	41.8 ± 11.4	42.2 ± 11.4	41.6 ± 11.3	41.7 ± 11.6
Body weight (kg) (mean ± SD)	78.1 ± 15.2	78.3 ± 15.6	76.2 ± 15.9	79.8 ± 14.1
BMI (kg/m^2^) (mean ± sd)	26.05 ± 4.37	26.24 ± 4.37	25.59 ± 4.44	26.33 ± 4.31
Viral load (copies/mL):*				
<10^6^	50.5%	52.5%	47.6%	51.6%
10^6^ to 2 × 10^6^	19.0%	20.5%	21.0%	15.6%
≥2 × 10^6^	30.2%	26.2%	31.5%	32.8%
HCV genotype 1 (%)†	6.7	4.9	8.9	6.3
HCV genotype 1a (%)†	7.8	10.7	7.3	5.5
HCV genotype 1b (%)†	58.6	58.2	59.7	57.8
HCV genotype 2 (%)†	2.4	3.3	2.4	1.6
HCV genotype 2a (%)†	1.9	2.5	2.4	0.8
HCV genotype 3 (%)†	20.9	16.4	18.5	27.3
Median ALT (U/L)	76.8 ± 49.0	80.7 ± 50.5	75.2 ± 49.3	74.5 ± 47.2
Necro-inflammation score (mean ± SD)	7.6 ± 2.5 (*n* = 326)	7.9 ± 2.5 (*n* = 109)	7.5 ± 2.5 (*n* = 107)	7.3 ± 2.6 (*n* = 110)
Fibrosis score (mean ± sd)	3.1 ± 1.8 (*n* = 324)	3.3 ± 1.7 (*n* = 108)	2.8 ± 1.8 (*n* = 107)	3.1 ± 1.8 (*n* = 109)

FAS: full analysis set, GL: glycyrrhizin

* HCV viral load was assessed by real-time PCR with the COBAS Ampliprep/Taqman-Systems (Roche Diagnostics) (detection limit: 15 IU/mL).

† HCV genotype was assessed by VERSANT HCV Genotype 2.0 Assay (Siemens Diagnostics).

The study was performed in compliance with the Declaration of Helsinki, ICH-Guidelines for Good Clinical Practice, and the applicable regional regulatory requirements of all countries involved in this study. Approval by the respective Ethics Committees and signed Informed Consent of each patient were obtained before starting the study. This study is referenced in the EudraCT database with the EudraCT number 2004-000773-60.

### Study endpoints

The primary efficacy endpoints were the proportion of patients with ≥50% ALT reduction after 12-weeks double-blind treatment and the proportion of patients with improvement of modified Histology Activity Index (modified HAI) [19], necro-inflammation score (decrease of ≥1) after 52 weeks compared to baseline. Secondary efficacy endpoints were mean change of ALT from baseline at the end of week 12, proportion of patients with ALT reduction below 1.5× ULN within the 12-weeks double-blind treatment period and after 52-weeks of treatment, proportion of patients with improvement in total HAI (decrease ≥1) and in fibrosis score (decrease ≥1), change in viral load determined by HCV-RNA-activity and change in quality of life (QOL) assessed through a validated local translation of the 36-item Health Survey developed at RAND (Research and Development Corp.) [20].

Laboratory tests were performed centrally by LKF (Laboratorium für Klinische Forschung GmbH, Schwentinental/Germany). Determination of HCV-RNA-activity and HCV-genotype was done by Labor Lademannbogen, Hamburg/Germany (formerly Labor Prof. Arndt & Partner). HCV viral load was assessed by real-time PCR with the COBAS Ampliprep/Taqman-Systems (Roche Diagnostics) (detection limit: 15 IU/mL). VERSANT HCV Genotype 2.0 Assay (Siemens Diagnostics) was used to determine HCV-genotype.

### Liver histology

Liver biopsy was taken from all patients during the 2 months screening period who gave informed consent. A second biopsy was done at the end of treatment (Week 52). All samples were assessed by three pathologists blinded to all clinical information (R.G., London/UK; T.R. Leuven/Belgium; H.D., Cologne/Germany).

A decrease of ≥1 was defined as improvement in the modified HAI, while an increase of ≥1 was defined as deterioration. No change in the HAI scores was defined as no further deterioration (see suppl. Data).

### Study drug and randomisation

The study drug used was Stronger Neo-Minophagen® C (SNMC, Minophagen Pharmaceutical Co. Ltd., Tokyo/Japan) in 20 mL ampoules each containing monoammonium glycyrrhizinate equivalent to 40 mg GL. At each administration, subjects received 5 ampoules of 20 mL of either study drug or placebo, or a combination of the two. Active study medication and placebo were not distinguishable.

### Statistical analysis

The detailed statistical analysis plans are provided in the online supplementary information.

## Results

### Biochemical response after 12 weeks double-blind phase

For the first primary endpoint the proportion of patients with ALT reduction ≥50% was significantly higher with 5×/week GL (28.7%, *P* < 0.0001) and 3×/week GL + 2×/week placebo (29.0%, *P* <0.0001) compared with 5×/week placebo (7.0%) after 12 weeks of treatment. Under active treatment, mean ± SD change from baseline in ALT values (95% confidence interval) was −32.8 ± 41.2 U/L (−40.2 to −25.4) and −26.9 ± 31.5 U/L (−32.5 to −21.2), respectively. The decrease occurred within 2 weeks. In contrast, under placebo mean ± sd ALT levels increased slightly by 0.6 U/L (−8.4 to 9.5). Detailed data on biochemical response is given in Table 2.

**Table 2:** Improvement of ALT levels after double-blind treatment (after 12 weeks) with glycyrrhizin (FAS)

Regimen	5×/week GL *N* = 122	3×/week GL + 2×/week Placebo *N* = 124	5×/week Placebo *N* = 128
Proportion of ALT reduction ≥50% (*N*, %)	35 (28.7%)* (*P* < 0.0001)	36 (29.0%)* (*P* < 0.0001)	9 (7.0%)
ALT mean change from baseline (U/L) (mean ± SD)	−32.8 ± 41.2* (*P* < 0.0001)	−26.9 ± 31.5* (*P* < 0.0001)	0.6 ± 51.0
<1.5× ULN† (*N*, %)	35 (28.7%)* (*P* < 0.0001)	23 (18.5%)* (*P* = 0.0031)	8 (6.3%)

FAS: full analysis set, GL: glycyrrhizin

* Significant difference.

† ULN = Upper Limit of Normal (normal range: male 0–22 U/L, female 0–17 U/L).

### Histological results after 52 weeks of treatment

Concerning the second primary endpoint improvement in modified HAI necro-inflammation score (decrease ≥1), the proportion of patients with an improvement (necrosis and inflammation) was 44.9% with 5×/week GL and 46.0% with 3×/week GL after 52 weeks of treatment. A significant number of patients showed no further deterioration in modified HAI necro-inflammation scores (18.4% with 5×/week GL; 15.0% 3×/week GL). Combined results for improved modified HAI necro-inflammation and/or no further deterioration are 63.3% (5×/week GL) and 61.0% (3×/week GL), respectively. Data on modified HAI necro-inflammation score after 52 weeks is given in Table 3 and suppl. data.

**Table 3:** Changes of necro-inflammation and fibrosis score after 52 weeks of treatment with glycyrrhizin in evaluable patients (*N*, %) (FAS, open phase treated patients only)

	Population (%)	5×/week GL	3×/week GL
Necro-inflammation	*N* = 249 (363)*	*N* = 136 (190)*	*N* = 113 (173)*
Improvement	113 (45.4%)	61 (44.9%)	52 (46.0%)
No change	42 (16.9%)	25 (18.4%)	17 (15.0%)
Deterioration	94 (37.8%)	50 (36.8%)	44 (38.9%)
Fibrosis	*N* = 249 (363)*	*N* = 137 (190)*	*N* = 112 (173)*
Improvement	83 (33.3%)	51 (37.2%)	32 (28.6%)
No change	84 (33.7%)	43 (31.4%)	41 (36.6%)
Deterioration	82 (33.0%)	43 (31.4%)	39 (34.8%)

FAS: full analysis set, GL: glycyrrhizin

()* Number of patients participating in the study

A decrease of ≥1 was defined as improvement in modified HAI, while an increase of ≥1 was defined as deterioration.

Regarding fibrosis score, improvement (decrease ≥1) was higher with 5×/week GL than with 3×/week GL (37.2% vs. 28.6%, respectively). However, this did not reach statistical significance (*P* > 0.05). No further deterioration was seen in 31.4% (5×/week GL) and 36.6% (3×/week GL) of patients, respectively. Combined results for improved HAI fibrosis and/or no further deterioration were 67.0% [33.3% with improvement and 33.7% with no further deterioration (see Table 3)].

### Secondary endpoint

Predefined secondary endpoint of the study was mean change of ALT from baseline at week 12. After the double-blind treatment phase ALT values were significantly improved in comparison with placebo, reaching one of the additional endpoints of this study. The proportion of patients with ALT ≥1.5× ULN at baseline and decrease in values <1.5× ULN after 12 weeks was significantly higher with 5×/week GL (28.7%) and with 3×/week GL + 2×/week placebo (18.5%) than with placebo (6.3%). An overview on changes in ALT levels after 12 weeks of treatment is given in Table 2.

Further decrease of ALT was observed during the open phase with the mean values −14.2 ± 33.2 U/L and −8.2 ± 37.5 U/L in the group treated with 5×/week and 3×/week GL, respectively.

**Fig. 3:**
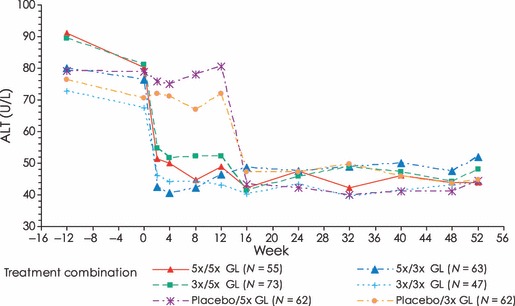
Time course of serum ALT-levels during the study. *N* Number of patients; *ALT* alanine aminotransferase, *GL* glycyrrhizin. ALT values for total dose were analysed by stratification of 6 sub-groups of all study regimens. Four sub-groups receiving GL from week 0 (5×/5× GL, 5×/3× GL, 3×/5× GL, 3×/3× GL) showed an immediate decrease in ALT values (mean) compared to the two placebo arms. There was no dose dependent effect on ALT reduction among the four groups receiving GL throughout the study assessed at week 12 and week 52 (reduction range: 76–96%). The two placebo arms showed subsequently a decrease in mean ALT levels once they were switched to either 5× GL/week or 3× GL/week in the open phase. After switching to GL, the response rate became similar to the other 4 sub-groups (81% under GL, 48% under placebo).

ALT values for total dose were also analysed by stratification of six sub-groups of all study regimens. The mean ALT levels of the six treatment groups are demonstrated in Fig. 3. Four sub-groups receiving GL throughout the study from week 0 showed an immediate decrease in ALT values compared to the two placebo arms. Obviously, there was no dose dependent effect among those groups on ALT reduction assessed at week 12 and week 52 (reduction range: 76–96%). The two placebo arms showed subsequently a decrease in mean ALT levels once they were switched to either 5× GL/week or 3× GL/week in the open phase. After switching to verum, the response rate became similar to the other four sub-groups (81% under GL, 48% under placebo) (Fig. 3).

### Serum HCV-RNA

No changes in serum HCV-RNA as measured by real-time PCR (i.e. in median HCV serum virus load) during the double-blind or open phase were observed. There were no differences among treatment groups (data not shown).

### Safety

Treatment with GL was well tolerated. Adverse events, laboratory parameters and vital signs congruently showed changes in line with pseudoaldosteronism and thus can be explained by the mechanism of action of GL. The most frequent adverse events possibly or probably related to the study drug were hypertension (including aggravated pre-existing hypertension), hypokalaemia, headache, paraesthesia, peripheral oedema, upper abdominal pain, increased blood creatine phosphokinase and nausea. A total of 4.2% patients in the double-blind phase and 6.6% patients in the open phase of patients dropped out due to treatment related adverse events, respectively. Table 4 summarises the most frequent adverse events related to GL observed during the double-blind phase. They appeared to be dose-dependent. Hypertension and hypokalaemia were more frequent during the open phase in patients receiving 5×/week GL than in patients receiving 3×/week GL. Paraesthesia occurred during the double-blind phase in the GL groups only. Systolic blood pressure (mean ± SD) changed in the three treatment groups (i.e. 5×/week GL, 3×/week GL and 5×/week placebo) during the double-blind phase by 3.5 ± 16.2 mmHg, 2.8 ± 14.7 mmHg and −0.5 ± 11.1 mmHg, respectively. The diastolic pressure increased by 0.8 ± 8.0 mmHg, 3.1 ± 9.1 and 0.6 ± 7.6 mmHg, respectively.

**Table 4:** Most frequent adverse events (AEs) possibly/probably related to glycyrrhizin during the 12 week double-blind treatment (*N*, %) (SAF)

	5×/week GL *N* = 123	3×/week GL *N* = 127	Placebo *N* = 129
Number of subjects	123	127	129
Number of subjects with AEs	57	48	35
Number of AEs	138	101	82
Relationship possible (*N*, %)			
Hypertension aggravated	12 (8.7%)	1 (1.0%)	4 (4.9%)
Hypertension NOS*	7 (5.1%)	4 (4.0%)	–
Headache	5 (3.6%)	6 (5.9%)	1 (1.2%)
Abdominal pain, upper	1 (0.7%)	2 (2.0%)	6 (7.3%)
Paraesthesia	2 (1.4%)	4 (4.0%)	–
Blood pressure increased	1 (0.7%)	3 (3.0%)	2 (2.4%)
Relationship probable (*N*, %)			
Hypertension NOS	6 (4.3%)	5 (5.0%)	–
Paraesthesia	5 (3.6%)	5 (5.0%)	–
Hypokalaemia	5 (3.6%)	3 (3.0%)	–

SAF: safety analysis set, GL: glycyrrhizin

* NOS: Not otherwise specified.

### Quality of life (QOL)

Slight improvements in QOL including health change assessments were observed during both treatment phases and in all treatment groups. In the double-blind phase, a more pronounced improvement was observed in patients receiving GL compared to placebo. In the open phase, patients treated more frequently with GL (5×/week) showed more distinct improvement in QOL than patients receiving 3×/week GL. However, no significant changes could be observed in either treatment phase. Details can be obtained from the online Table S1 in the supplementary file.

## Discussion

This study demonstrates that treatment with GL reduces ALT levels. After the 12 week double-blind treatment phase ALT values were significantly improved in comparison to placebo. Suppression of necro-inflammation as well as improvement of fibrosis was observed in patients treated with GL either 5×/week or 3×/week for 52 weeks. Despite the fact that no changes in serum HCV-RNA levels were seen during the double-blind or open phase, GL leads to a decrease in ALT values compared to the two placebo arms.

The combination of peg-IFN and RBV has been the standard of care (SOC) for patients with chronic hepatitis C since 2001. However, certain patient populations in particular non-responders to prior IFN-based therapies show very poor response rates to these therapies with less than 16% of patients clearing HCV RNA [21,22]. Thus, alternative options are needed. Treatment that could halt or diminish the progression of fibrosis would be beneficial. However, three major studies, HALT-C, EPIC-3 and COPILOT, failed to show efficacy of low dose pegylated interferon on fibrosis progression in patients who previously failed to IFN based therapies [5,23].

Innovative agents that are in clinical development include HCV protease, HCV polymerase and HCV NS 5A inhibitors as well as the host targeting agent (HTA) alisporivir [24]. Currently, phase III study results of two HCV protease inhibitors are available [25,26]. They have to be combined with peg-IFN + RBV and therefore there are no solutions for IFN intolerant patients at the moment. Two phase III studies, REALIZE and RESPOND-2, explored the benefits of telaprevir and boceprevir in previous peg-IFN + RBV failure patients, respectively [6,25]. Response rates were higher among patients who had previously relapsed than those who were non-responders, partial as well as null responders [27]. Still more than half of non-responder patients will fail to upcoming triple therapies with first generation HCV protease inhibitors and response rates are particular poor for subjects with advanced fibrosis or cirrhosis, HCV genotype 1a and African Americans. Treatment regimens with all oral anti-HCV therapies combining protease inhibitors, polymerase inhibitors as well as NS 5A inhibitors are in earlier stages of clinical development [28]. As a remaining alternative out of the currently available therapies, GL is considered worth re-evaluating its utility in the treatment of IFN non-responder or IFN non-tolerant chronic hepatitis patients.

The result of this study suggests that GL may be helpful in patients who did not respond to previous IFN based therapies or who cannot tolerate interferon. GL led to a significant reduction of ALT levels in comparison to placebo after 12 weeks of treatment. Further decrease of ALT was observed during the 40 weeks of open phase. First experience with GL in IFN non-responders in European chronic hepatitis C patients was reported by Schalm *et al.* (2003) [29]. The recent study was performed to provide data from a larger European patient population and to assess the effect of GL on ALT and on liver histology. ALT is a marker of inflammatory activity in the liver [30]. Moriyama *et al.* (2005) reported that patients achieving ALT levels less than twice the ULN after IFN therapy had a reduced risk of progression to HCC [31]. Several studies have shown that long-term treatment with GL decreases elevated ALT levels in chronic hepatitis, while development of HCC was reduced [15]. Rino *et al.* (2006) demonstrated that consistent reduction of elevated ALT level with GL significantly decreased the risk of developing HCC from 66% to 41% [32].

Necro-inflammation and fibrosis scores improved significantly after GL treatment. Combining the results for improved modified HAI grade and no further deterioration, histological efficacy of GL reaches 63.3% (5×/week GL) and 61% (3×/week GL), respectively. In regard to fibrosis scores, the combined results reached 67%. However, a control group has not been part of the study design since two biopsies 52 weeks apart in peg-IFN plus ribavirin non-responders receiving placebo for 52 weeks was not regarded ethical.

Necro-inflammation scores were improved although they did not reach the targeted endpoints of ≥60% improvement. A closer look at the results of necro-inflammation sub-scores confirmed the positive effects of GL. In particular, the results of periportal or periseptal interface hepatitis scores and the focal (spotty) lytic necrosis, apoptosis, and focal inflammation indicate a favorable inhibition. No such effects have been demonstrated in IFN non-responders before [3]. When this study was planned, there were no reliable histological data available on non-responders. An improvement rate of 60% was considered feasible (by assumption) based on a limited source of information available, but in hindsight this proved too optimistic [33], particularly in IFN non-responding, difficult-to-treat patients.

The treatment rationale for hepatitis C should include not only reduction in liver disease progression and HCC development but also an improvement in health-related quality of life (HRQOL). HRQOL was significantly decreased in patients with chronic hepatitis C with advanced fibrosis and cirrhosis and treatment with peg-IFN + RBV improved HRQOL as assessed by SF-36 questionnaire [34]. In our study, HRQOL-improvements were observed in both study phases, exceeded placebo effect in patients receiving GL and appeared to be dose dependent. Despite the reported negative impact of long-term intravenous application on QOL, a good compliance of GL therapy was confirmed by low drop-out rates. Based on the mode of action of GL, adverse events, laboratory parameters and vital signs were compatible with symptoms of pseudoaldosteronism. While the incidence of AEs was slightly higher than that observed in the past clinical studies [10,11] treatment with GL was found generally well tolerated and this study confirmed the safety profile of GL.

Limitations of this study need to be considered. No long-term follow-up of patients was performed to investigate if the potential beneficial effects on disease activity had also a long-term impact on progression of liver disease. This information would be required to determine the optimal treatment duration of GL also considering cost-benefit ratios. Moreover, treatment with GL required frequent intravenous injections over a prolonged period of time and thus might be feasible in clinical practice only in some countries.

In conclusion, it was shown that GL reduces ALT and prevents disease progression in a proportion of chronic hepatitis C patients who did not respond to or tolerate previous IFN based therapies. Improvement of ALT levels in IFN non-responders and IFN intolerant patients irrespective of HCV-RNA can be achieved in a proportion of patients. Since frequent i.v. injection (5×/week or 3×/week) required for a GL therapy can possibly be a burden for chronic hepatitis patients, development of a formulation which allows less frequent administration or non-parenteral application would be a significant step forward.

## Abbreviations

HCV: hepatitis C virus
SVR: sustained virological response
HCC: hepatocellular carcinoma
IFNα: interferon alpha
peg-IFNα: pegylated interferon alpha
RBV: ribavirin
RNA: ribonucleic acid
DAA: direct antiviral agents
GL: glycyrrhizin
CHC: chronic hepatitis C
ALT: alanine aminotransferase
ULN: upper limit of normal
HAI: histology activity index
PCR: polymerase chain reaction
SNMC: Stronger Neo-Minophagen® C
QOL: quality of life
FAS: full analysis set
PPS: per protocol set
LOCF: last-observation-carried-forward
RAND: Research and Development Corp. (Santa Monica/USA)
SAF: safety analysis set
SD: standard deviation
HRQOL: Health-related QOL

## SUPPORTING INFORMATION

**Table S1:** Patient characteristics (FAS).

**Table S3.1:** Periportal or periseptal interfacehepatitis (piecemeal necrosis) score in patients (*N*, %)after 52 weeks of treatment (all patients treated during theopen phase).

**Table S3.2:** Confluent necrosis score inpatients (*N*, %) after 52 weeks of treatment (allpatients treated during the open phase).

**Table S3.3:** Focal (spotty) lytic necrosis,apoptosis, and focal inflammation score in in patients (*N*,%) after 52 weeks of treatment (all patients treated duringthe open phase).

**Table S3.4:** Portal inflammation score in inpatients (*N*, %) after 52 weeks of treatment (allpatients treated during the open phase).
