# Supplementary material for: Glycyrrhizin in patients who failed previous interferon alpha-based therapies: biochemical and histological effects after 52 weeks
Source: J Viral Hepat. 2012 Aug;19(8):537–46. doi: 10.1111/j.1365-2893.2011.01579.x (PMC3584517; doi:10.1111/j.1365-2893.2011.01579.x)
Supplement: Supplementary file 1 [file jvh0019-0537-SD1.doc]

**Tables**

Table 1: Patient characteristics (FAS)

| **Characteristics** | **All patients** | **5x/week GL** | **3x/week GL + 2x/week Placebo** | **5x/week Placebo** |
| --- | --- | --- | --- | --- |
| **Total number of patients** | **n=374** | **n=122** | **n=124** | **n=128** |
| **Male (%)** | 67.9 | 64.8 | 63.7 | 75.0 |
| **Age (year) (mean ± SD)** | 41.8 ± 11.4 | 42.2 ± 11.4 | 41.6 ± 11.3 | 41.7 ± 11.6 |
| **Body weight (kg) (mean ± SD)** | 78.1 ± 15.2 | 78.3 ± 15.6 | 76.2 ± 15.9 | 79.8 ± 14.1 |
| **BMI (kg/m^2^) (mean ± SD)** | 26.05 ± 4.37 | 26.24 ± 4.37 | 25.59 ± 4.44 | 26.33 ± 4.31 |
| **Viral load (copies/mL):* < 10^6^ 10^6^ - 2x10^6^ ≥ 2x10^6^** | 50.5% 19.0% 30.2% | 52.5% 20.5% 26.2% | 47.6% 21.0% 31.5% | 51.6% 15.6% 32.8% |
| **HCV genotype 1 (%)**** | 6.7 | 4.9 | 8.9 | 6.3 |
| **HCV genotype 1a (%)**** | 7.8 | 10.7 | 7.3 | 5.5 |
| **HCV genotype 1b (%)**** | 58.6 | 58.2 | 59.7 | 57.8 |
| **HCV genotype 2 (%)**** | 2.4 | 3.3 | 2.4 | 1.6 |
| **HCV genotype 2a (%)**** | 1.9 | 2.5 | 2.4 | 0.8 |
| **HCV genotype 3 (%)**** | 20.9 | 16.4 | 18.5 | 27.3 |
| **Median ALT (U/L)** | 76.8 ± 49.0 | 80.7 ± 50.5 | 75.2 ± 49.3 | 74.5 ± 47.2 |
| **Necro-inflammation score (mean ± SD)** | 7.6 ± 2.5 (n=326) | 7.9 ± 2.5 (n=109) | 7.5 ± 2.5 (n=107) | 7.3 ± 2.6 (n=110) |
| **Fibrosis score (mean ± SD)** | 3.1 ± 1.8 (n=324) | 3.3 ± 1.7 (n=108) | 2.8 ± 1.8 (n=107) | 3.1 ± 1.8 (n=109) |

FAS: full analysis set, GL: glycyrrhizin
*HCV viral load was assessed by real-time PCR with the COBAS Ampliprep/Taqman-Systems (Roche Diagnostics) (detection limit: 15 IU/mL).
**HCV genotype was assessed by VERSANT HCV Genotype 2.0 Assay (Siemens Diagnostics).
