# Supplementary material for: Glycyrrhizin in patients who failed previous interferon alpha-based therapies: biochemical and histological effects after 52 weeks
Source: J Viral Hepat. 2012 Aug;19(8):537–46. doi: 10.1111/j.1365-2893.2011.01579.x (PMC3584517; doi:10.1111/j.1365-2893.2011.01579.x)
Supplement: Supplementary file 3 [file jvh0019-0537-SD3.doc]

Supplemental data:

Table 3.1: Periportal or periseptal interface hepatitis (piecemeal necrosis) score in patients (N, %) after 52 weeks of treatment (all patients treated during the open phase)

| All patients 5X/week SNMC 3X/week SNMC |
| --- |
| N=363(249)* N=190(136)* N=173(113)* |
| Improvement by 4 points 1 (0.4%) --- 1 (0.9%) |
| Improvement by 3 points 4 (1.6%) 2 (1.5%) 2 (1.8%) |
| Improvement by 2 points 23 (9.2%) 15(11.0%) 8(7.1%) |
| Improvement by one point 58 (23.3%) 28(20.6%) 30(26.6%) |
| No change 98 (39.4%) 57 (41.9%) 41(36.3%) |
| Worsening by one point 43 (17.3%) 23 (16.9%) 20 (17.7%) |
| Worsening by 2 points 19 (7.6%) 11 (8.1%) 8 (7.1%) |
| Worsening by 3 points 3 (1.2%) --- 3 (2.7%) |
| No remarks 114 (-) 54 (-) 60 (-) |

( )* Patients with evaluable values

Table 3.2 : Confluent necrosis score in patients (N, %) after 52 weeks of treatment (all patients treated during the open phase)

| All patients 5X/week SNMC 3X/week SNMC |
| --- |
| N=363(249)* N=190(136)* N=173(113)* |
| Improvement by 4 points 1 (0.4%) --- 1 (0.9%) |
| Improvement by 3 ppoints 2 (0.8%) 2 (1.5%) --- |
| Improvement by 2 points 13 (5.2%) 6(4.4%) 7(6.2%) |
| Improvement by one point 19 (7.6%) 9(6.6%) 10(8.9%) |
| No change 173 (47.7%) 95 (69.9%) 78(69.0%) |
| Worsening by one point 28 (11.3%) 16 (11.8%) 12 (10.6%) |
| Worsening by 2 points 12 (4.8%) 7 (5.2%) 5 (4.4%) |
| Worsening by 4 points 1 (0.4%) 1 (0.7%) --- |
| No remarks 114 (-) 54 (-) 60 (-) |

( )* Patients with evaluable values

Table 3.3: Focal (spotty) lytic necrosis, apoptosis, and focal inflammation score in

in patients (N, %) after 52 weeks of treatment (all patients treated during the open phase)

| All patients 5X/week SNMC 3X/week SNMC |
| --- |
| N=363(249)* N=190(136)* N=173(113)* |
| Improvement by 2 points 6 (2.4%) 4(2.9%) 2(1.8%) |
| Improvement by one point 64 (25.7%) 34(25.0%) 30(26.6%) |
| No change 119 (47.8%) 63 (46.3%) 56(49.6%) |
| Worsening by one point 54 (21.7%) 30 (22.1%) 24 (21.2%) |
| Worsening by 2 points 5 (2.0%) 4 (2.9%) 1 (0.9%) |
| Worsening by 3 points 1 (0.4%) 1 (0.7%) --- |
| No remarks 114 (-) 54 (-) 60 (-) |

( )* Patients with evaluable values

Table 3.4: Portal inflammation score in in patients (N, %) after 52 weeks of treatment (all patients treated during the open phase)

| All patients 5X/week SNMC 3X/week SNMC |
| --- |
| N=363(249)* N=190(136)* N=173(113)* |
| Improvement by 3 ppoints 3 (1.2%) 2 (1.5%) 1 (0.9%) |
| Improvement by 2 points 25 (10.0%) 16(11.8%) 9(8.0%) |
| Improvement by one point 65 (26.1%) 37(27.2%) 28(24.8%) |
| No change 105(42.2%) 54 (39.7%) 51(45.1%) |
| Worsening by one point 46 (18.5%) 25 (18.4%) 21 (18.6%) |
| Worsening by 2 points 5 (2.0%) 2 (1.5%) 3 (2.7%) |
| No remarks 114 (-) 54 (-) 60 (-) |

( )* Patients with evaluable values
